# *Journal of Applied Crystallography* welcomes eight new Co-editors

**DOI:** 10.1107/S160057672500072X

**Published:** 2025-02-01

**Authors:** Janos Hajdu, Garry J. McIntyre, Flora Meilleur

**Affiliations:** a The European Extreme Light Infrastructure ERIC, Za Radnici 835, 25241Dolní Břežany, Czech Republic; b Laboratory of Molecular Biophysics, Department of Cell and Molecular Biology Uppsala University Husargatan 3 UppsalaSE-75124 Sweden; c Australian Nuclear Science and Technology Organisation Lucas HeightsNSW2234 Australia; d Department of Molecular and Structural Biochemistry North Carolina State University Raleigh NC27695 USA; e Neutron Scattering Division Oak Ridge National Laboratory Oak Ridge TN37831 USA

**Keywords:** Co-editors, applied crystallography, structural science

## Abstract

The newest eight members of the Editorial Board of *Journal of Applied Crystallography* are introduced.

As with all IUCr journals, the Editorial Board of *Journal of Applied Crystallography* evolves as members complete their terms and the field of applied crystallography in its broadest sense expands.

Our most recent editorial on the scope of the journal introduced Louise Dawe (Wilfrid Laurier University, Waterloo, Ontario, Canada) as an additional Teaching and Education Editor (Dawe *et al.* 2022 ▸). Since then, three new Co-editors, Sabrina Disch (University of Duisburg-Essen, Germany), Jozef Keckes (Montanuniversität Leoben, Austria) and Parthapratim Munshi (Shiv Nadar Institution of Eminence, Delhi, India), have joined the Editorial Board, usually to replace retiring members.

The current scope of *Journal of Applied Crystallography* is

Many research topics in condensed matter research, materials science and the life sciences make use of crystallographic methods to study crystalline and non-crystalline matter with neutrons, X-rays and electrons. Articles published in *Journal of Applied Crystallography* focus on these methods and their use in identifying structural and diffusion-controlled phase transformations, structure–property relationships, structural changes of defects, interfaces and surfaces, *etc*. Developments of instrumentation and crystallographic apparatus, theory and interpretation, numerical analysis, and other related subjects are also covered. The journal is the primary place where crystallographic computer program information is published.

The scope of the journal is thus quite broad and continues to expand as new applications of crystallography or new techniques within crystallography and structural science emerge. A notable recent example is artificial intelligence, as exemplified by the virtual collection published early last year (Ekeberg, 2024 ▸). The broad scope of the journal is also shown by the larger number of submissions compared with the other IUCr journals, which has greatly increased the workload of each member of the Editorial Board in recent years.

In this issue, we are delighted to welcome eight new Co-editors (Fig. 1 ▸) to *Journal of Applied Crystallography* to help cover the growing number of submissions and the ever-broadening scope of articles in applied crystallography and structural science that we aim to publish.

Professor René Guinebretière is a member of the Institut de Recherche sur les Céramiques, IRCER UMR CNRS, of the University of Limoges, France. His field of research is materials physics (materials science in general) and the associated X-ray characterization techniques, with expertise in ceramics, defects, diffuse scattering, X-ray diffraction and X-ray optics. He carries out his research mainly on synchrotron radiation sources.

Dr James K. Harper is a Research Consultant at US Synthetic and an Adjunct Associate Professor in the Department of Chemistry of the University of Utah, Salt Lake City, Utah, USA. He brings to the Editorial Board a much desired expertise in NMR with extensive knowledge of its relevance to crystallography and complementarity to diffraction techniques. He also brings a valuable industrial connection in his present consultancy role with US Synthetic, a major producer of synthetic diamond tools for oil and gas companies.

Professor Venkatesha R. Hathwar is an Assistant Professor and Programme Director of Physics in the School of Physical and Applied Sciences at Goa University, India. He has expertise in single-crystal and powder X-ray diffraction, neutron diffraction, Rietveld refinement, charge-density analysis using high-resolution X-ray data, synchrotron data, and high-pressure data. He is presently applying this expertise to the study of photo-luminescent and photo-catalytic materials, phase transitions, and hybrid functional materials for energy applications. He also has expertise in the analysis of weak intermolecular interactions and structure–property correlations using quantum crystallographic methods.

Professor Dr Karolina Jurkiewicz is a faculty member in the August Chełkowski Institute of Physics of the University of Silesia in Katowice, Poland. She works in materials science with research interests in topics related to X-ray characterization of the atomic-scale, molecular and supramolecular structure of non-crystalline materials, nanomaterials, and disordered and amorphous-like phases, specifically structure–synthesis–property relationships in functional materials, extreme-pressure diffraction synchrotron studies and structural transformation of materials. She also has experience in synchrotron X-ray diffraction and pair-distribution-function analysis as well as computer simulations and structure modeling.

Dr Thomas J. Lane leads the Photobiology Group (PBIO) of the Center for Free-Electron Laser Science (CFEL) at DESY in Hamburg, Germany. He has expertise in time-resolved crystallography and artificial intelligence applications for protein structures, and he has worked with the industry to apply this knowledge to advance drug discovery.

Professor Adrian Mancuso is currently the Director of Physical Sciences at Diamond Light Source in Didcot, UK. He has expertise in methods and instrumentation at XFEL and synchrotron facilities across a range of disciplines, focused mainly on exploiting spatially coherent X-rays to observe the structure and dynamics of matter.

Dr Florian Meneau is the manager of the Cateretê beamline for the Coherent Scattering Group in the Brazilian Center for Research in Energy and Materials (CNPEM) at the Brazilian Synchrotron Light Laboratory (LNLS) in Campinas, Brazil. His main research interests lie in synchrotron techniques and instrumentation. He is already well versed in the demands of editing, having served as one of the Guest Editors of the SAS2022 special issue of *Journal of Applied Crystallography.*

Professor Katharine Page is an Assistant Professor in the Materials Science and Engineering Department of the University of Tennessee, Knoxville, USA, and a Joint Faculty Member in the Neutron Scattering Division at Oak Ridge National Laboratory in Oak Ridge, Tennessee, USA. She has broad experience in neutron scattering science, facilities and techniques, with expertise in materials chemistry and structural science over a wide range of complex material systems of technological interest, especially energy materials.

With the expanded Editorial Board, nearly all IUCr geographical regions will be represented, and the range of expertise needed to address the interests of cutting-edge applied crystallographic and structural science research will be significantly enhanced. We look forward to working with all Co-editors, both ongoing and new, to increase the impact and reach of *Journal of Applied Crystallography.* Short biographies of all the Editors and Co-editors can be found by clicking on their photographs on the editor web page: https://journals.iucr.org/j/services/editors.html.

To close, we thank the recently retired Co-editors, Trevor Forsyth (Lund University, Sweden), Václav Holý (Charles University in Prague, Czech Republic), Dhananjai Pandey (Indian Institute of Technology, Varanasi, India), Arwen Pearson (University of Hamburg, Germany) and Dmitri Svergun (EMBL, Hamburg, Germany) for their invaluable contributions to the Editorial Board over many years.

## Figures and Tables

**Figure 1:**
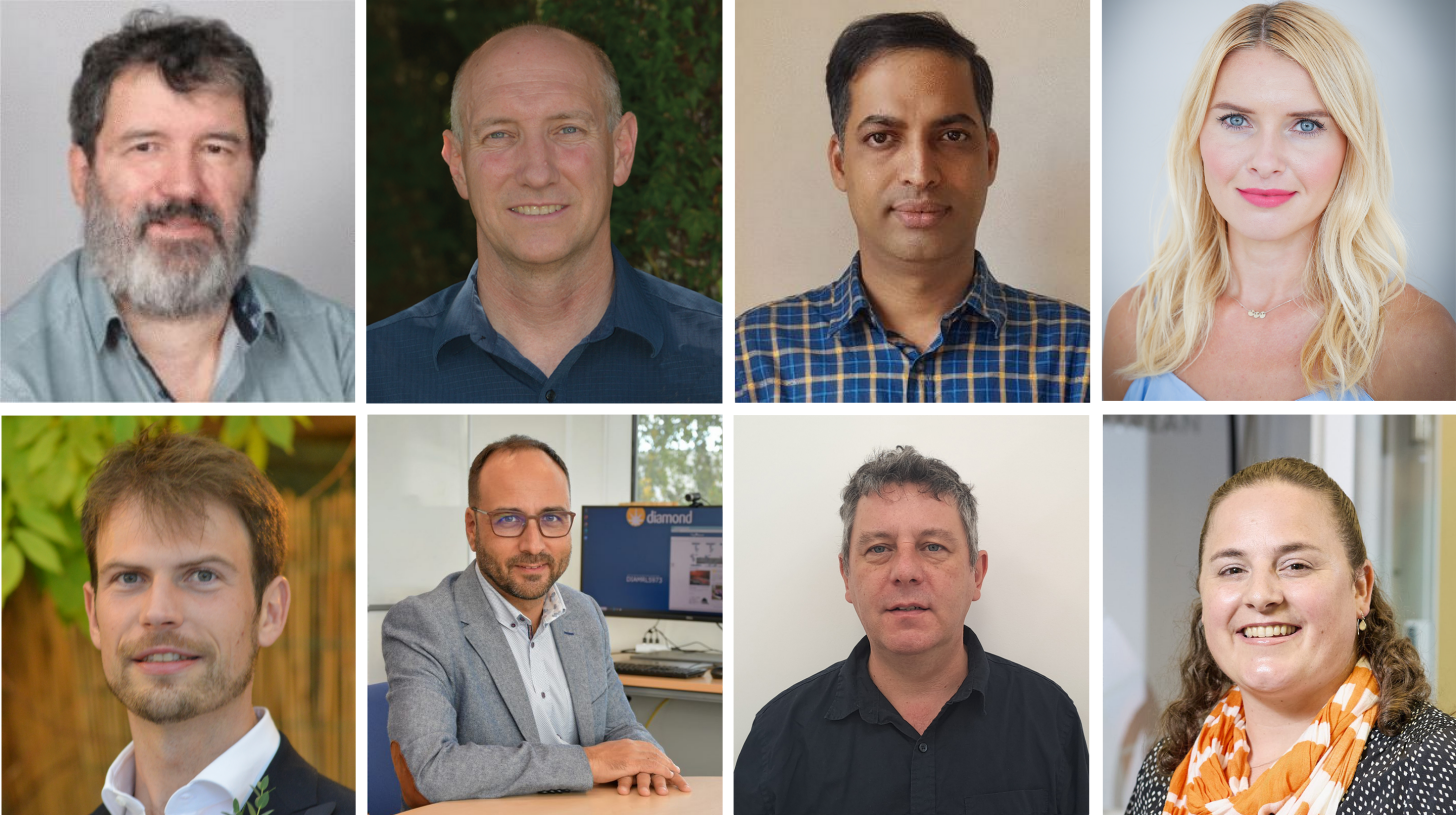
Our new Co-editors René Guinebretière, James Harper, Venkatesha Hathwar, Karolina Jurkiewicz, Thomas Lane, Adrian Mancuso, Florian Meneau and Katherine Page.
